# Hypertriglyceridemic waist phenotype indicates insulin resistance in adolescents: validation study front hyperglycemic clamp-Brazilian Metabolic Syndrome Study (BRAMS)

**DOI:** 10.1186/1758-5996-7-S1-A145

**Published:** 2015-11-11

**Authors:** Francieli Barreiro Ribeiro, Cleliani de Cássia da Silva, Ana Carolina Junqueira Vasques, Mariana Porto Zambon, Ana Maria De Bernardi Rodrigues, Daniella Fernandes Camilo, Maria Ângela Reis de Góes Monteiro Antonio, Bruno Geloneze Neto, Francieli Barreiro Ribeiro, Cleliani de Cássia da Silva, Ana Carolina Junqueira Vasques, Mariana Porto Zambon, Ana Maria De Bernardi Rodrigues, Daniella Fernandes Camilo, Maria Ângela Reis de Góes Monteiro Antonio, Bruno Geloneze Neto

**Affiliations:** 1 UNICAMP, Campinas, Brazil

## Background

The increase in levels of triglycerides (TG) and waist circumference (WC) characterize the phenotype of hypertriglyceridemic waist (HTGW phenotype) that predicts cardiovascular risk in adults to join the atherogenic triad: hyperinsulinemia, high concentrations of Apo -B and small dense LDL particles. Recent studies have demonstrated that metabolic syndrome (MS) is a marker and cardiovascular alterations in adolescents.

## Objective

To investigate the association of HTGW phenotype with metabolic syndrome (MS) and insulin resistance (IR) assessed by hyperglycemic clamp in adolescents.

## Materials and methods

Cross-sectional study with 847 adolescents (10-19 yrs., 537 girls). It assessed pubertal stage (self-assessment-Tanner), anthropometric and laboratory parameters. IR assessed by HOMA-IR method and the hyperglycemic clamp (glucose infusion rate adjusted for lean mass [TIGMM]) n=77; IR considered HOMA-IR> 75th percentile HTGW Phenotype defined by the presence of TG> 100 mg/dL and dc> 75 cm in pubertal girls (sensitivity (S) 71.2% and (E) specificity 61.8%), CC> 84 cm in post-pubertal girls (S: 84.7% and E 63%), WC> 86 cm in pubescent boys (S 72.9% and 80.7% E) and WC >94 cm in post-pubescent boys (85.5% S and E 82.4%). Cutoff points for CC determined by ROC curves, considering the value of a larger sum of S and E to IR identification, stratified by gender and pubertal stage.

## Results

The phenotype (HTGW phenotype versus healthy) was associated with the highest average total cholesterol (183±33 vs 151±27), LDL (111±30 vs 88±24), systolic blood pressure (119±13 vs 109±13), diastolic pressure (76±8 vs 69±10), HOMA-IR index (4.7±2.9 vs. 1.8±1.1), insulin (13±21.8 vs 9±5); Glucose (87±10 vs 81±10) and lower HDL levels (42±11 vs 51±11) compared to healthy; p <0.001.

## Conclusion

The Results demonstrate the usefulness of the phenotype of hypertriglyceridemic waist identify the presence of insulin resistance and metabolic syndrome in adolescents' population in plan and in clinical practice.

**Figure 1:**
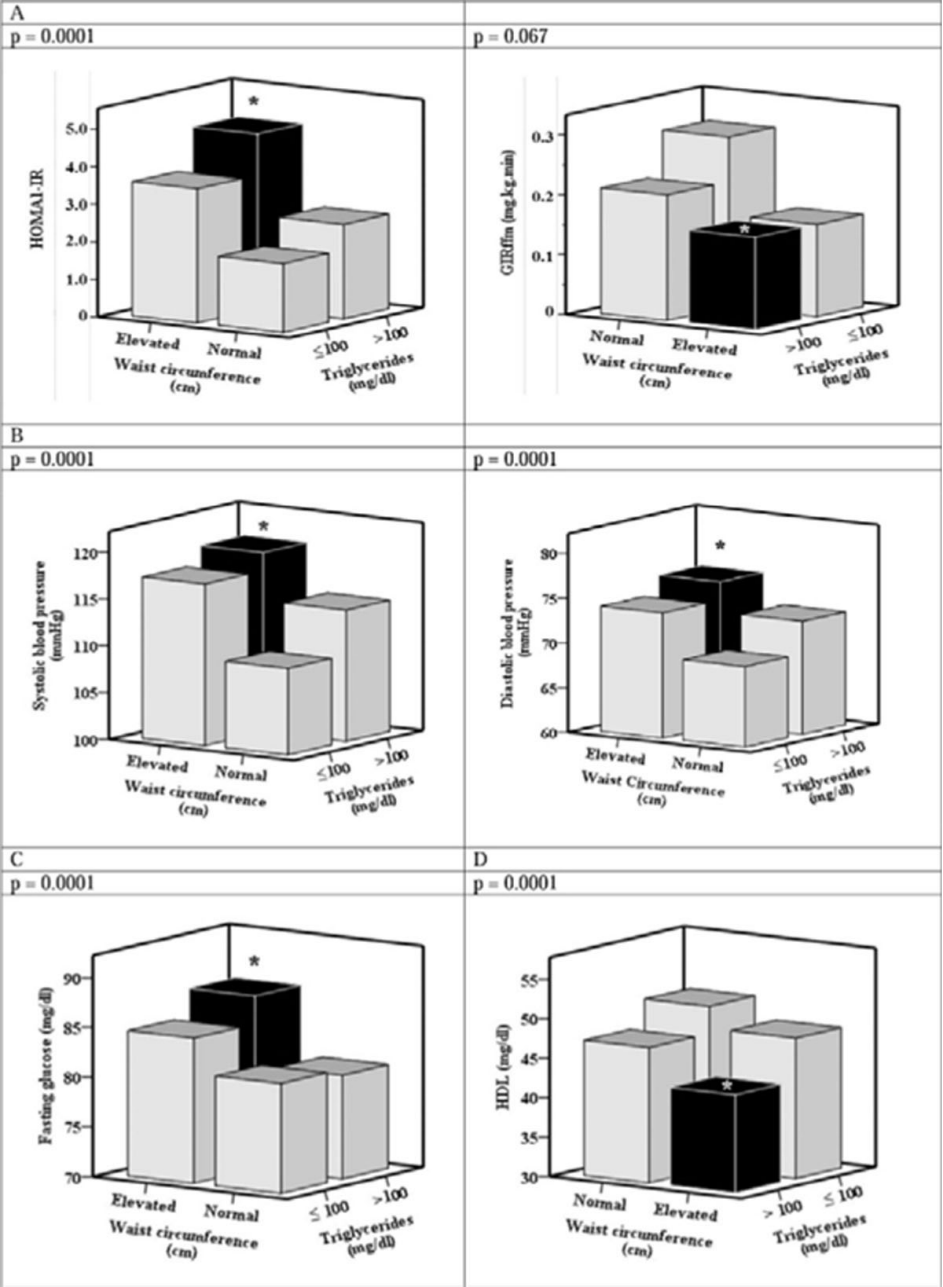
Distribution of the components of the metabolic syndrome in the presence of the Hypertriglyceridemic waist phenotype.

**Figure 2:**
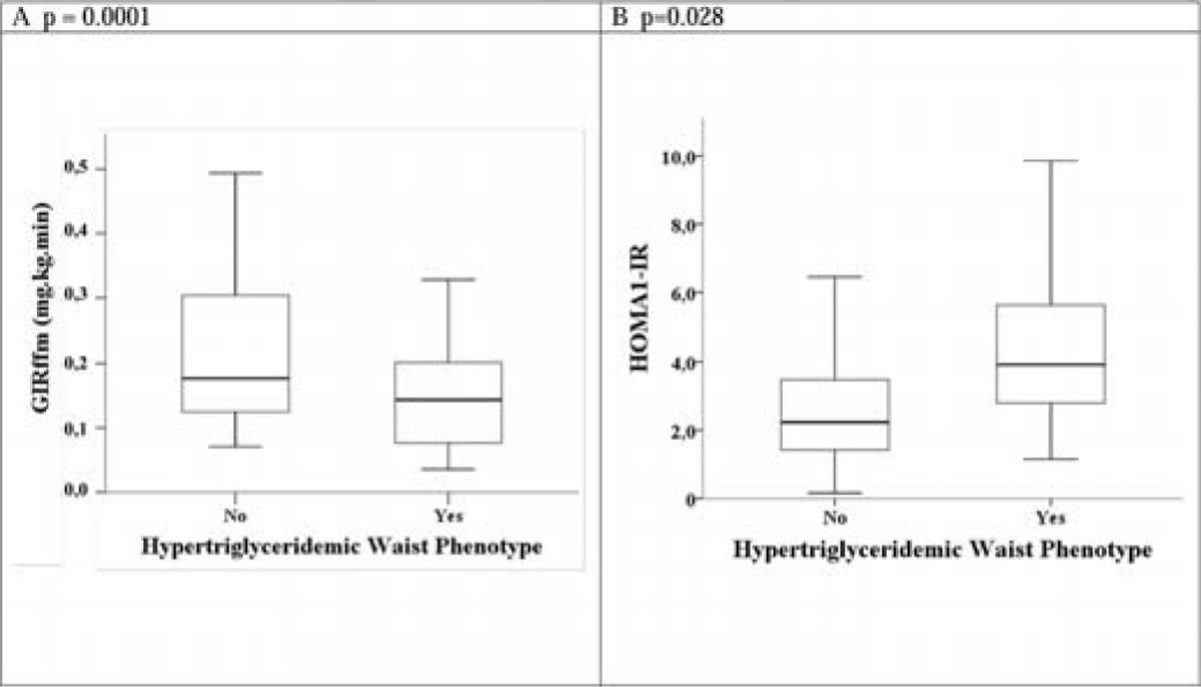
Hypertriglyceridemic waist phenotype compared in the presence of IR assessed by HOMA-IR index and hyperglycemic clamp.

